# The phenotypic heterogeneity of obese and nonobese patients with severe asthma and comparison of omalizumab–mepolizumab treatment efficiency in these patients

**DOI:** 10.1097/MD.0000000000035247

**Published:** 2023-10-27

**Authors:** Şeyma Özden, Fatma Merve Tepetam, Cihan Örçen, Tuğçe Yakut

**Affiliations:** a Department of Immunology and Allergy, University of Health Sciences, Süreyyapaşa Chest Diseases and Thoracic Surgery Training and Research Hospital, Istanbul, Turkey; b University of Health Sciences, Derince Training and Research Hospital, Kocaeli, Turkey; c Department of Immunology and Allergy, University of Health Sciences, Diyarbakir Gazi Yaşargil Training and Research Hospital, Diyarbakir, Turkey.

**Keywords:** endotype, mepolizumab, obese asthma, obesity, omalizumab, phenotype, severe asthma

## Abstract

In obese severe asthmatics, the degree of type 2 inflammation may vary according to their atopic status and past smoking history. In this study, we aimed to analyze the clinical and physiopathological features of obese and nonobese severe asthmatics treated with omalizumab or mepolizumab treatment. In addition we aimed to compare the clinical, spirometric outcomes and total peripheral eosinophilic count (TEC) changes after treatment with these 2 biologic agents in obese and nonobese groups. In this retrospective, cross sectional study, 121 severe asthmatic treated with biologic agents (omalizumab = 88 or mepolizumab = 33) for at least 16 weeks were included. Obese (n: 44) and nonobese severe asthmatics (n: 77) were analyzed according to whether they provided a ≥ 10 pack/years (p/y) or <10 p/y smoking history and were found to be atopic. Obese and nonobese groups were compared in terms of the change in the asthma control test, asthma attacks, TEC, and forced expiratory volume in the first second (FEV_1_) after treatment. In patients with ≥10 p/y smoking history, nonobese group had a significantly higher TEC compared to obese group [median (min–max) 660 cells/μL (200–1500) vs 300 cells/μL (110–770); p: 0.013]. Within the nonobese group, nonatopic patients had a significantly higher TEC compared to atopic patients [median (min–max) 1200 cells/μL (100–2100) vs 310 cells/μL (0–2730); p: 0.021]. Both biologic agents had similar effects on improving asthma control test and in reducing asthma attacks; however, mepolizumab was more effective in suppressing TEC. The improvement in FEV_1_ in obese group following biologic 2 agents was very similar but in nonobese group, mepolizumab was found to be superior (510 mL vs. 295 mL; p: 0.034). In our real-life study, nonobese severe asthmatics with ≥10 p/y smoking history and those that were nonatopic had higher TEC. Compared to omalizumab, mepolizumab was superior at reducing TEC in all asthmatics and in improving FEV_1_ in nonobese group.

## 1. Introduction

The worldwide prevalence of overweight and obesity has nearly tripled over the past 4 decades, and represents one of the most serious unmet public health challenges of the 21st century.^[1]^ Obesity is a risk factor for many life-threatening conditions such as atherosclerosis, hypertension and type 2 diabetes mellitus. Obesity has also been proven to be a major risk factor for asthma.^[2]^ Asthma with obese patients is usually severe and difficult to control.^[3]^ Although severe asthma accounts for only 10% of all asthma cases, the severe asthma patient group accounts for 60% of the total medical cost of asthma treatment.^[3,4]^ Considering that up to 60% of patients with severe asthma are obese, it is obvious that this is a major health problem and socio-economic burden.^[5]^ The obese asthma phenotype varies both clinically and physiologically depending upon whether asthma develops before or after the onset of obesity. Asthma with subsequent obesity is characterized by early-onset disease (typically < 12 years of age), elevated markers of allergic inflammation (atopy, allergic symptoms, elevated serum immunoglobulin E level), more severe physiological changes (airway obstruction and hypersensitivity) whereas asthma developing after obesity is characterized by a later onset disease (≥12 years of age), female dominance, less allergic inflammation, less airflow obstruction, and less hypersensitivity.^[6]^

Although the underlying pathobiological mechanism of obese asthmatic patients is associated with non-type 2 inflammation and many groups have reported decreased sputum eosinophils with increasing body mass index (BMI), increased eosinophil count was detected in the airway wall.^[5,7,8]^ Total peripheral eosinophil count (TEC) may vary depending on whether the patients have atopy or ≥10 pack/year (p/y) smoking history (≥10 p/y).

In fact, in many cluster analysis studies, atopic asthma has been associated with reduced TEC.^[9–11]^ In the Severe Asthma Research Program study 58% of asthmatics included in the late-onset, nonatopic eosinophilic phenotype were obese.^[10]^ However, in the unbiased biomarkers for the prediction of respiratory disease outcomes study, the cluster 2 group, which included ≥10 p/y smoking history 41% of whom were obese, had the highest TEC [301 cells/μL (119–560)]. Cluster 4, a more atopic group, 56% [200 cells/μL (99.7–385)] of whom were obese, had only moderately TEC and frequent exacerbations.^[11]^ Thus, in obese severe asthmatics, the degree of type 2 inflammation may vary according to their atopic status and past smoking history.

Current biological agents used to treat severe asthma mainly target type 2 inflammation.^[12,13]^ Published studies suggest that omalizumab gives a less positive response in obese compared to nonobese asthmatics.^[14]^ Data on the responses of obese patients to mepolizumab treatment have not been published yet except post hoc analyzes. In this study, we compared the efficacy of omalizumab and mepolizumab in obese and nonobese asthmatics. All patients received a biological agent for a minimum of 16 weeks. Both between and within the obese and nonobese groups, we looked at the effect of the 2 biological agents regarding the change in clinical improvement, spirometric measurements and TEC.

## 2. Method

### 2.1. Study design

In this retrospective study we analyzed the response of severe asthmatics to 1 of 2 biological agents, omalizumab or mepolizumab, based upon their clinical and pathophysiological features, including the presence or absence of obesity. Three centers (Süreyyapaşa Chest Diseases and Thoracic Surgery Training and Research Hospital, Istanbul; Derince Training and Research Hospital, Kocaeli and Diyarbakir Gazi Yaşargil Training and Research Hospital, Diyarbakir) were included in this study. The study protocol was approved by the local ethics committee of University of Health Sciences, Süreyyapaşa Chest Diseases and Thoracic Surgery Training and Research Hospital (Approval identification number: 202). Patients with comorbid diseases such as malignancy, rheumatological disease, bronchiectasis, vasculitis, sarcoidosis, bronchopulmonary aspergillosis or interstitial lung disease, HIV and pregnancy were excluded from the study. The data was collected from electronic or paper hospital medical records and consisted of all medical care received between 2012 and 2021.

### 2.2. Patients

Patients’ asthma diagnosis was confirmed according to the criteria specified in the global initiative for asthma guidelines.^[15]^ To be included in the study, patients had to have asthma that could not be controlled on high-dose inhaled corticosteroids therapy and a second asthma control agent; have had 2 or more asthma exacerbations over the previous year requiring systemic steroids for a minimum of 3 days; or have had an asthma exacerbation requiring hospitalization over the previous 1 year. All patients were required to have been compliant with all prescribed inhaler medications, to have demonstrated correct inhaler technique, and to have had optimization of concomitant diseases. If all of these conditions were met, the patient was considered to have severe asthma. All patients diagnosed with severe asthma were required to complete a 3-month trial of montelukast or a long-acting muscarinic antagonist. Those who failed to achieve good asthma control were started on omalizumab or mepolizumab.

Among these patients; patients over 18 years of age with a total immunoglobulin (Ig) E level of 30–1500 IU/mL, who had perennial allergen sensitivity as determined by specific IgE (ImmunoCap; Pharmacia Diagnostics AB, Uppsala, Sweden) or by skin prick test, were started on Omalizumab (Xolair, Novartis, Basel, Switzerland) therapy for at least 16 weeks. Omalizumab treatment dose and dose intervals were determined based on the patient’s weight and baseline total IgE level. Patients with peripheral blood eosinophil level ≥ 150 cells/μL while on systemic steroid therapy or ≥300 cells/μL during the previous year were started on mepolizumab 100 mg (Nucala, Glaxosmithkline, London, United Kingdom) therapy for at least 16 weeks. Mepolizumab was preferred in patients who were candidate for both biologics, with the presence of nasal polyps and peripheral eosinophil predominance. According to the definition of the World Health Organization (WHO), patients with a BMI ≥ 30 kg/m^2^ are classified as obese, while patients with a BMI < 30 kg/m^2^ were classified nonobese.^[16]^

### 2.3. Collected data

Demographic characteristics of patients, age of asthma onset, duration of disease, smoking history, presence of atopy, BMI, asthma control test (ACT) scores, number of asthma attacks, forced expiratory volume in the first second (FEV_1_), forced expiratory flow between 25% and 75% of forced vital capacity (FEF_25–75_) values (mL) and percentages, TEC, total IgE levels and duration of treatment with biological agents were recorded. Although a total of 180 patients were examined, 121 patients were included in the study because 59 patients were excluded due to missing data. 32 patients with missing spirometrics parameters and 27 patients with missing other parameters (smoking history, asthma onset age, etc) were excluded from the study.

### 2.4. Subset analysis of obese and nonobese patients

Obese and nonobese asthmatics were classified according to their smoking history;

- First group: Smoking history ≥10 p/y.
- Second group: Smoking history <10 p/y or never smoker.

Than patients with ≥10 p/y smoking history were excluded and remaining obese and nonobese asthmatics were classified according to presence of atopy.

- First group: Presence of atopy.
- Second group: Absence of atopy.

In both analyses, intragroup TEC, spirometric measurements and numbers of asthma attacks were compared.

### 2.5. Assessment of omalizumab–mepolizumab treatment efficacy

The efficacy of the biological agents was assessed using the ACT, numbers of asthma attacks, FEV_1_ (mL), FEF_25/75_ (%), and changes in TEC were evaluated after omalizumab and mepolizumab treatments.

### 2.6. Comparison of treatment efficacy in obese and nonobese groups considering both biological agents

Obese and nonobese patients were compared in terms of change in ACT, number of asthma attacks, TEC, and FEV_1_ after treatment with both biological agents.

### 2.7. The rates of responder patients according to eosinophilic status of obese and nonobese patient groups

The obese and nonobese patient groups were each subdivided into 2 groups based upon their baseline TEC (≥300 cells/μL or <300 cells/μL). The treatment response of these 4 groups to the 2 biologics was determined based upon the change from baseline in ACT, number of asthma attacks and FEV_1_. This constituted 3 responder groups: ACT responders, asthma attack responders, and FEV_1_ responders. They are defined as follows:

1. ACT responder: A patient with an increase of ≥3 points in ACT or an ACT score of ≥20 points or higher.
2. Asthma attack responder: A patient who had a decrease of more than 50% in the number of attacks.
3. FEV_1_ responder: A patient who had an increase of more than 100 mL or an increase of 10%.

### 2.8. Statistical analysis

Statistical analyzes were performed using SPSS software (version 21.0 for Windows; SPSS Inc., Chicago, IL). Parametric variables were presented as means and standard deviations, nonparametric variables as medians and minimum–maximum (min–max). Number of cases and percentages were used for categorical variables. Chi-square test was used in the analysis of categorical variables. Whether the continuous variables were normally distributed or not was determined by Kolmogorov–Smirnov and histogram analysis. Normally distributed numerical variables were analyzed using an independent sample *t*-test. Mann–Whitney *U*-test were used to compare numerical variables that did not show normal distribution. The different clinical and biological markers before and after treatment were evaluated by the paired sample *t*-test if normal distributed, and by the Wilcoxon signed-rank test if not normally distributed. Statistical significance was accepted when the 2-sided *P*-value was <.05.

## 3. Results

When 121 adult patients with severe asthma from a total of 3 centers, including 55 from Istanbul, 44 from Kocaeli and 22 from Diyarbakir, were evaluated retrospectively, 44 patients (36.4%) were obese. Most of the patients were women (82.6%), and the mean age of all patients was 48.45 ± 12.50 years.

### 3.1. Analysis of obese and nonobese patients

While the mean age of obese severe asthmatic is higher than nonobese severe asthmatics. (52.84 ± 10.31 years vs. 45.94 ± 13.00 years; p: 0.003), other demographic characteristics such as sex, age of onset of asthma, disease duration, smoking history, clinical features such as presence of atopy, asthma control level, and frequency of attacks, as well as biophysiological features such as TEC, FEV_1_, and FEF_25–75_ were similar. But atopy rate and TEC tended to be higher in nonobese severe asthmatics. Most of the patients in both groups used omalizumab as a biologic agent (obese group: 75.0%, nonobese group: 71.4%), and there was no difference between the groups in terms of treatment duration (Table 1).

**Table 1:** Demographic and clinical characteristics of patients.

	Obese n: 44	Nonobese n: 77	*P*-value
Age (years old), mean ± SD	52.84 ± 10.318	45.94 ± 13.005	.003*
Sex, female, n	40	60	.118+
Asthma onset age (years old), mean ± SD	33.57 ± 12.785	30.14 ± 13.740	.179*
Asthma duration time (years), median (min–max)	20.00 (4–44)	15.00 (4–213)	.092‡
Time of treatment with biological agent (months), median (min–max)	32.00 (4–99)	27.00 (4–91)	.274‡
Comorbidities			
Nasal polyps, n (%)	6 (13.6)	21 (27.3)	.132+
GER, n (%)	15 (34.1)	15 (19.5)	.116+
Rhinitis, n (%)	36 (81.8)	63 (81.8)	>.05+
Smoking history, n (%)			
Current smoker	1 (0.8)	6 (5.0)	
Ex smoker	16 (13.2)	26 (21.5)	.503+
Nonsmoker	27 (22.3)	45 (37.2)	
Presence of atopy, n (%)	37 (30.6)	63 (52.1)	.946+
ACT, median (min–max)	11.00 (5–19)	12.00 (5–18)	.140‡
Asthma attack, mean ± SD	6.48 ± 3.695	6.23 ± 3.211	.978*
FEV_1_ (mL) mean ± SD	1818.79 ± 583.024	1924.11 ± 749.359	.486*
FEV_1_ (%) mean ± SD	68.88 ± 18.526	69.02 ± 20.007	.975*
FEF_25–75_ (mL) mean ± SD	17,004.52 ± 678.70	1780.23 ± 917.58	.698*
FEF_25–75_ (%) mean ± SD	52.84 ± 22.56	50.66 ± 23.26	.686*
TEC (cells/µL), median (min–max)	335.00 (0–2800)	410.00 (0–2730)	.237‡
Total IgE (IU/mL), median (min–max)	395.00 (36–1644)	224.00 (15–2447)	.060‡
Omalizumab/mepolizumab, n (%)	33 (75.0)/11 (25.0)	55 (71.4)/22 (28.6)	.832

ACT = asthma control test, GER = gastroesophageal reflux, TEC = total peripheral eosinophil count.

* Independent samples *t*-test.

+Chi square test.

‡ Mann–Whitney *U* test.

### 3.2. Phenotypic analyses according to the presence of obesity and smoking history

The severe asthmatics were first divided into 2-groups as obese and nonobese. Later both groups were evaluated according to whether they had ≥10 p/y or <10 p/y smoking history. When patients with ≥10 p/y and <10 p/y smoking history were compared; TEC, FEV_1_ (mL), FEF_25–75_ (%) levels and number of attacks were similar according to their smoking history (Table 2a). But the TEC of the nonobese group with ≥10 p/y smoking history was significantly higher than the obese group with ≥10 p/y smoking history [median (min–max) 660 cells/μL (200–1500) vs 300 cells/μL (110–770); *P* = .013].

After we excluded ≥10 p/y severe asthmatics and compared the obese and nonobese severe asthmatics according to atopy status only in the nonobese group, TEC of nonatopic patients was statistically significantly higher than that of atopic patients [median (min–max) 1200 cells/μL (100–2100) vs 310 cells/μL (0–2730)), p: 0.021] (Table 2b).

### 3.3. Assessment of omalizumab and mepolizumab treatment efficacy

Patients who received omalizumab had a longer duration of treatment that did those who received mepolizumab (47.35 ± 26.090 months and 12.39 ± 6.946 months, respectively p: <0.001). Regardless of which biologic was taken, there was a significant improvement in ACT, a reduced number of asthma attacks, an improvement in FEV_1_ and FEF_25–75_, and a reduced TEC (Table 3).

**Table 3:** Assessment of omalizumab and mepolizumab treatment efficacy.

Treatment duration (months) mean ± SD	Omalizumab n: 88			Mepolizumab n: 33		
	47.35 ± 26.090			12.39 ± 6.946)		*P*-value < .001‡
	Pretreatment	Posttreatment	*P*-value	Pretreatment	Posttreatment	*P*-value
ACT, median (min–max)	11.00 (5–19)	22.00 (12–25)	<.001*	12.00 (6–18)	23.00 (20–25)	<.001*
Asthma attack, median (min–max)	6.00 (1–20)	0.0 (0–4)	<.001*	5.00 (2–12)	0.00 (0–3)	<.001*
FEV_1_ (mL) mean ± SD	1835.87 ± 681.97	2195.71 ± 698.97	<.001+	2002.57 ± 731.17	2576.52 ± 620.40	<.001+
FEF_25–75 (_%) mean ± SD	52.13 ± 23.72	70.61 ± 25.21	<.001*	50.10 ± 20.66	68.05 ± 18.3	<.001*
TEC (cells/µL), median (min–max)	300.00 (0–1800)	165.00 (0–1600)	<.001*	990.00 (120–2800)	100.00 (0–1130)	<.001*

TEC: total peripheral eosinophil count.

* Wilcoxon *t*-test.

+Paired samples *t*-test.

‡ Independent sample *t*-test.

### 3.4. Comparison of treatment efficacy in obese and nonobese groups considering both biological agents

**Figure 1.**
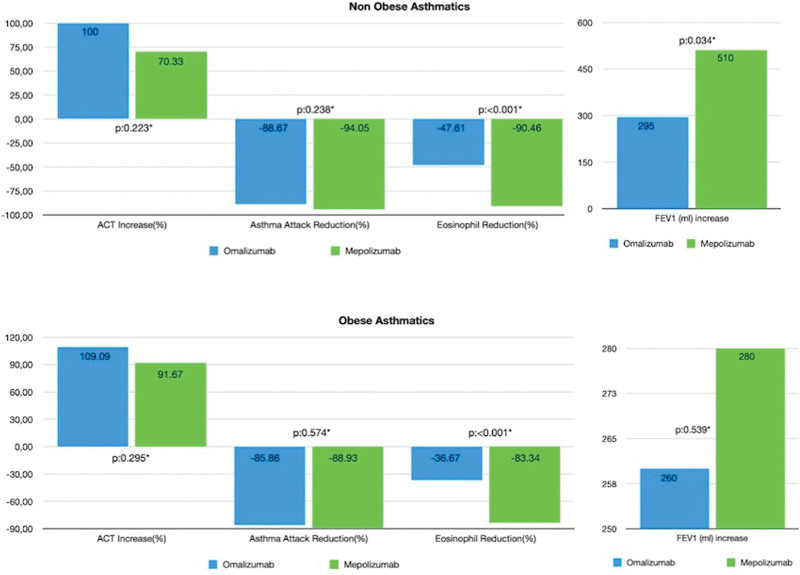
Comparison of treatment efficacy in obese and nonobese groups considering both biological agents.

The improvement in ACT score and reduced number of asthma attacks did not differ regardless of the biological agent taken in either the obese or nonobese severe asthmatics. However, mepolizumab produced a greater reduction in the TEC in both the obese and nonobese groups compared to omalizumab. Mepolizumab also produced a greater increase in FEV_1_ but only in the nonobese group (Fig. 1). When all asthmatics were evaluated regardless of which biological agent they were treated with, there was no statistically significant difference between the increase in FEV_1_ between obese and nonobese severe asthmatics patients (in nonobese group: 330.00 mL (25.00–1710.00), in obese group: 270.00 mL (5.00–1100.00; p: 0.429).

### 3.5. Comparison of percentage of responder patients according to eosinophilic status (≥300 cells/μL and < 300 cells/μL) in obese and nonobese severe asthmatics

**Figure 2.**
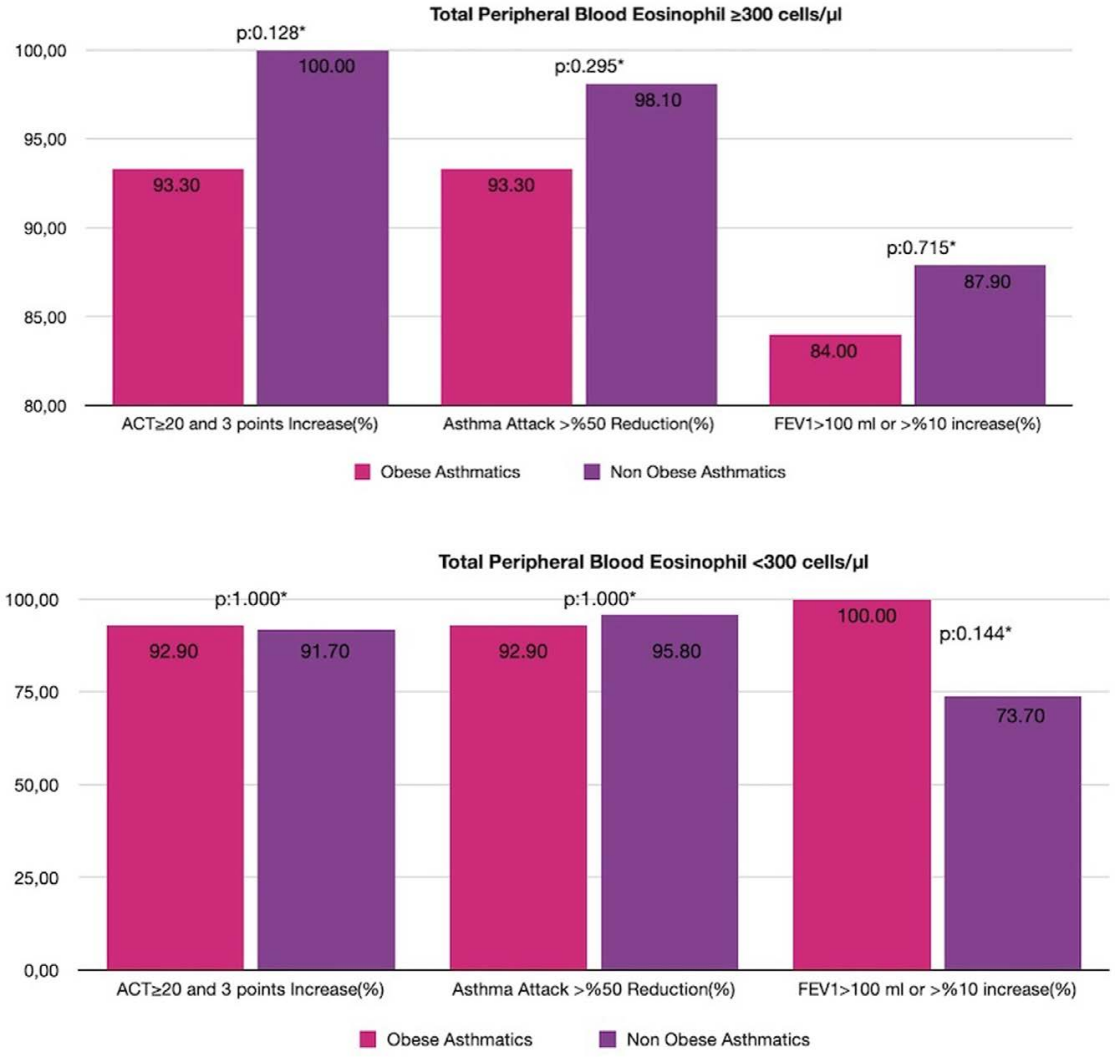
Comparison of percentage of responder patients according to the total peripheral eosinophil count in obese and nonobese groups.

The percentage of ACT responders, asthma attack responders, and FEV_1_ responders, as defined in the method section, within both the obese and nonobese groups did not differ significantly regardless of whether they had a baseline TEC that was ≥300 cells/μL or < 300 cells/μL (Fig. 2).

## 4. Discussion

In our 3-center study in which severely asthmatic patients treated with omalizumab or mepolizumab were evaluated retrospectively, obese asthmatics accounted for 36.4% of these patients. We found that severe obese asthmatic patients were older which was in good agreement with previous results reported in the literature.^[12,17]^ However, contrary to previous studies, we cannot say that severe obese asthmatic patients had poorer asthma control, had a history of more frequent attacks or were associated with greater lung function impairment compared to severe nonobese asthmatics.^[3,18–20]^

The patients were analyzed by considering the presence of atopy and history of heavy smoking, which may contribute to the phenotypic heterogeneity of both obese and nonobese severe asthmatics. In our study, patients with ≥ 10 p/y smoking history had a higher TEC if they were in the nonobese group vs the obese group. Furthermore, only in the nonobese groups the TEC was higher in the nonatopic compared to the atopic asthmatics. While many clustering studies have shown that the presence of atopy and obesity are associated with lower TEC,^[9–11]^ our study showed that the 2 conditions (obesity and atopy) overlap. And so, in obese severe asthmatics atopy status have not effect TEC.

Moreover, patients with ≥10 p/y smoking history weren’t included in the cluster analysis, except for the unbiased biomarkers for the prediction of respiratory disease outcomes study.^[11]^ In this study cluster 2 group, which included ≥10 p/y smoking history, 41% of whom were obese, had the highest TEC. But in our study, we analyzed the patients according to the presence of obesity, which is the confounder factor. We found that in nonobese asthmatics, a history of ≥10 p/y smoking was associated with a higher TEC; but this did not reach statistical significance (Table 2a). However, nonobese asthmatics with <10 p/y smoking history who were non atopic had a statistically higher TEC than those who were atopic (Table 2b).

In our study, the onset of asthma occurred in adulthood mostly and therefore the obese asthmatic group likely have had obesity prior to the development of asthma; however, since this information is obtained from the anamnesis of the patients, it gives the information that asthma symptoms worsen rather than the age of onset of asthma. This phenotype, as previously discussed, is less severe than early onset asthma which is then complicated by obesity.^[21]^

Following treatment with omalizumab and mepolizumab, we saw an improvement in ACT, a decrease in the number of asthma attacks, an increase in spirometric values, and a reduction in TEC which are consistent with real-life data.^[22–24]^ There is a limited number of studies evaluating biological treatment efficacy in obese asthmatics patients. In a recent study, with 26 weeks of omalizumab treatment, a significant improvement in asthma control was found in both obese and nonobese patients; however, those who remained uncontrolled at the end of treatment had a higher BMI than those who had improved asthma control.^[14]^ Our study findings are in agreement.

The association of BMI to the degree of FEV_1_ response to biologics has varied based upon the individual study. In our study improvement of FEV_1_ after biological treatment was in favor of nonobese asthmatics, although it was not statistically significant (in nonobese group: 330.00 mL (25.00–1710.00), in obese group: 270.00 mL (5.00–1100.00; p: 0.429). However, when asthmatics were evaluated in regards of biological preference (omalizumab and mepolizumab), FEV_1_ response to omalizumab was similar in both obese and nonobese groups, but the response to mepolizumab was much greater in the nonobese asthmatics (Fig. 1). Similarly, in the post hoc analysis of the mepolizumab as adjunctive therapy in patients with severe asthma and efficacy of mepolizumab add-on therapy on health-related quality of life and markers of asthma control in severe eosinophilic asthma studies, the FEV_1_ response to mepolizumab was less in patients weighing >90 kg.^[25]^ Likewise, when considering the FEV_1_ increase following treatment with omalizumab or mepolizumab, it still is unsettled as if there is any significant difference in response. In a meta-analysis of randomized controlled trials, the FEV_1_ increase was approximately 100 mL in both omalizumab and mepolizumab treated patients.^[26,27]^ However, in view of real life data, the change in FEV1 was above 200 mL for omalizumab and above 300 mL for mepolizumab.^[22,28]^ In other words, according to the reported data, approximately a 100 mL greater increase in FEV_1_ for mepolizumab compared to omalizumab.

When comparing omalizumab with mepolizumab in terms of clinic parameters in severe asthmatics not well controlled with high dose inhaled corticosteroids, a 2018 network meta-analysis found them to have similar efficacy in terms of improvement in asthma control while the increase in quality of life was evaluated in favor of omalizumab.^[26]^ In another systematic review on randomized controlled trials, mepolizumab treatment was found to show superior success in reducing the attacks in either treatment (trial population), rather than the overlap population that was a candidate for both biological agents.^[29]^ In our study, the efficacy of omalizumab and mepolizumab treatments were compared in obese and nonobese patients for the first time, and the clinical efficacy was found to be similar between the two biological treatments in both groups. However, though not statistically significant, the improvement in ACT was in favor of omalizumab treatment and the decrease in attacks was in favor of mepolizumab in both groups.

TEC is a biomarker used to determine the efficacy of treatment with both biological agents; there are conflicting studies on whether biologic agents are more effective in highly eosinophilic patients (≥300cells/μL). One of the most important reasons for the confusion in these studies is the evaluation of different parameters ranging from the global evaluation of treatment effectiveness scale to the spirometric change in evaluating the efficacy of the treatment, and another issue is the confounders, such as worsening of the parameters (number of attacks, ACT, etc) evaluated at baseline in the compared arm.^[30–32]^ In our study, when the patients in the obese and nonobese groups were compared at baseline, asthma control, number of attacks, and FEV_1_ values were similar at baseline, the percentages of responder patients according to all these parameters, is similar too when the high and low eosinophilic groups are considered separately.

Our study has several limitations. It is retrospective, thus at risk of bias. The mepolizumab group had fewer patients who were treated for a shorter period of time than the omalizumab group. However, our multicenter study drew attention to confounders such as atopy, smoking history, which may affect TEC. According to our current knowledge, this is the first study to indirectly compare the outcomes of omalizumab and mepolizumab treatment in obese and nonobese severe asthmatics.

## 5. Conclusion

Obese and nonobese asthma phenotypes have heterogeneity within themselves. ≥10 p/y smoking history is associated with higher TEC if they were in nonobese group vs. the obese group. Whereas only in nonobese group the presence of atopy is associated with lower TEC versus absence of atopy. However, atopy and ≥10 p/y smoking history do not significantly affect the TEC in obese severe asthmatics. Omalizumab and mepolizumab produced similar improvement in ACT score and in reducing the number of asthma attacks in both the obese and nonobese groups. However, mepolizumab produced a greater reduction in the TEC in both of these groups compared to omalizumab. Compared to omalizumab, mepolizumab resulted in a greater increase in FEV_1_ only in the nonobese group. Randomized controlled head-to-head comparative studies with larger numbers of patients are needed to provide more precise information.

**Table 2a:** Analysis of patients according to smoking history.

	Obese asthmatics			Nonobese asthmatics		
	≥10 pack/years smoking history n: 10	<10 pack/years smoking history n: 34	*P*-value	≥10 pack/years smoking history n: 10	<10 pack/years smoking history n: 34	*P*-value
TEC (cells/µL), median (min–max)	300.00 (100–770)	400.00 (0–2800)	.121*	660.00‡ (200–1500)	400.00 (0–2730)	.279*
FEV_1_ (mL), mean ± SD	1727.50 ± 435.52	1846.88 ± 626.17	.620+	2012.22 ± 1010.60	1907.23 ± 701.41	0.704+
FEF_25–75 (_%), mean ± SD	53.00 ± 25.51	52.78 ± 22.06	.982+	46.25 ± 23.25	51.64 ± 23.36	0.558+
Asthma attack, median (min–max)	6.00 (3–20)	5.00 (2–15)	.534*	6.00 (3–12)	6.00 (1–20)	0.990*

TEC: total peripheral eosinophil count.

*Mann–Whitney *U* test.

+Independent sample *t* test.

‡ When obese and nonobese patients were analyzed ≥ 10 p/y or < 10 p/y smoking history, the baseline eosinophil level of patients with a heavy smoking history in the nonobese group was significantly higher than in the obese group. [Median:660 (min: 200–max: 1500) vs median:300 (min: 110–max: 770); *P* = .013, Mann–Whitney *U* test.]

**Table 2b:** A total of 94 patients with a history of smoking < 10 p/y were evaluated according to their atopy status.

	Obese asthmatics			Nonobese asthmatics		
	Presence of atopy n: 29	Absence of atopy n: 5	*P*-value	Presence of atopy n: 51	Absence of atopy n: 9	*P*-value
TEC (cells/µL) median (min–max)	400.00 (0–2800)	1100.00 (200–2310)	.295*	310.00 (0–2730)	1200.00 (100–2100)	.021*
FEV_1_ (mL), mean ± SD	1782.68 ± 563.17	2200.00 ± 922.42	.227+	1962.86 ± 699.56	1440.00 ± 579.83	.116+
FEF_25–75 (_%), mean ± SD	50.84 ± 22.34	62.00 ± 20.9	.370+	52.61 ± 23.50	41.88 ± 20.64	.240+
Asthma attack median (min–max)	5.00 (2–15)	5.00 (3–8)	.814*	6.00 (1–20)	4.00 (3–12)	.136*

TEC = total peripheral eosinophil count.

*Mann–Whitney *U* test.

+Independent sample *t*-test.

## Author contributions

**Conceptualization:** Şeyma Özden, Cihan Örçen, Tuğçe Yakut.

**Data curation:** Fatma Merve Tepetam, Tuğçe Yakut.

**Formal analysis:** Fatma Merve Tepetam.

**Methodology:** Fatma Merve Tepetam, Cihan Örçen.

**Project administration:** Cihan Örçen, Tuğçe Yakut.

**Writing – original draft:** Şeyma Özden, Fatma Merve Tepetam, Cihan Örçen.

**Writing – review & editing:** Şeyma Özden.
